# Whole transcriptome analysis of HCT-8 cells infected by *Cryptosporidium parvum*

**DOI:** 10.1186/s13071-022-05565-4

**Published:** 2022-11-24

**Authors:** Lulu Sun, Juanfeng Li, Fujie Xie, Shanbo Wu, Tianren Shao, Xiaoying Li, Junqiang Li, Fuchun Jian, Sumei Zhang, Changshen Ning, Longxian Zhang, Rongjun Wang

**Affiliations:** 1 College of Veterinary Medicine, Henan Agricultural University, Zhengzhou, 450046 China; 2 International Joint Research Laboratory for Zoonotic Diseases of Henan, Zhengzhou, 450046 China

**Keywords:** *Cryptosporidium parvum*, HCT-8 cells, RNA-Seq, Whole transcriptome, ceRNA, Non-coding RNAs

## Abstract

**Background:**

*Cryptosporidium* species are zoonotic protozoans that are important causes of diarrhoeal disease in both humans and animals. Non-coding RNAs (ncRNAs) play an important role in the innate immune defense against *Cryptosporidium* infection, but the underlying molecular mechanisms in the interaction between human ileocecal adenocarcinoma (HCT-8) cells and *Cryptosporidium* species have not been entirely revealed.

**Methods:**

The expression profiles of messenger RNAs (mRNAs), long non-coding RNAs (lncRNAs), microRNAs (miRNAs) and circular RNAs (circRNAs) in the early phase of infection of HCT-8 cells with *Cryptosporidium parvum* and at 3 and 12 h post infection were analyzed using the RNA-sequencing technique. The biological functions of differentially expressed RNAs (dif-RNAs) were discovered through Gene Ontology (GO) and Kyoto Encyclopedia of Genes and Genomes (KEGG) enrichment analyses. The targeting relationships between three ncRNAs and mRNAs were analyzed using bioinformatics methods, followed by building a competing endogenous RNA (ceRNA) regulatory network centered on miRNAs.

**Results:**

After strictly filtering the raw data, our analysis revealed 393 dif-lncRNAs, 69 dif-miRNAs and 115 dif-mRNAs at 3 hpi, and 450 dif-lncRNAs, 129 dif-miRNAs, 117 dif-mRNAs and one dif-circRNA at 12 hpi. Of these, 94 dif-lncRNAs, 24 dif-miRNAs and 22 dif-mRNAs were detected at both post-infection time points. Eleven dif-lncRNAs, seven dif-miRNAs, eight dif-mRNAs and one circRNA were randomly selected and confirmed using the quantitative real-time PCR. Bioinformatics analyses showed that the dif-mRNAs were significantly enriched in nutritional absorption, metabolic processes and metabolism-related pathways, while the dif-lncRNAs were mainly involved in the pathways related to the infection and pathogenicity of *C. parvum* (e.g. tight junction protein) and immune-related pathways (e.g. cell adhesion molecules). In contrast, dif-miRNAs and dif-circRNA were significantly enriched in apoptosis and apoptosis-related pathways. Among the downregulated RNAs, the miRNAs has-miR-324-3p and hsa-miR-3127-5p appear to be crucial miRNAs which could negatively regulate circRNA, lncRNA and mRNA.

**Conclusions:**

The whole transcriptome profiles of HCT-8 cells infected with *C. parvum* were obtained in this study. The results of the GO and KEGG pathway analyses suggest significant roles for these dif-RNAs during the course of *C. parvum* infection. A ceRNA regulation network containing miRNA at its center was constructed for the first time, with hsa-miR-324-3p and hsa-miR-3127-5p being the crucial miRNAs. These findings provide novel insights into the responses of human intestinal epithelial cells to *C. parvum* infection.

**Graphical Abstract:**

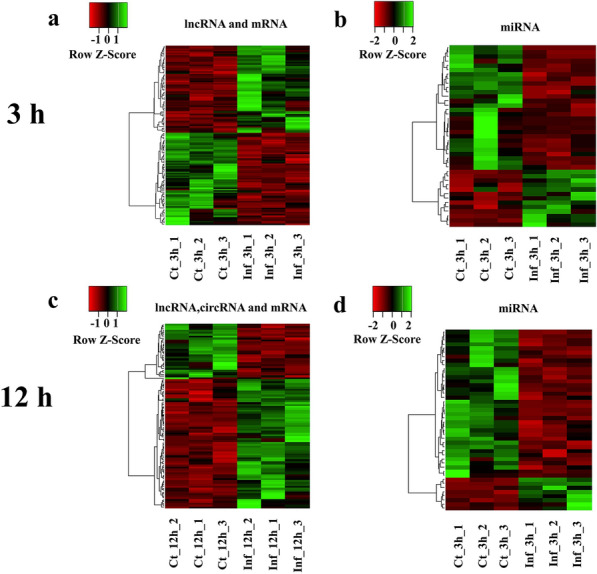

**Supplementary Information:**

The online version contains supplementary material available at 10.1186/s13071-022-05565-4.

## Background

*Cryptosporidium* species are intracellular gastrointestinal parasites that can infect the gastrointestinal epithelium of vertebrate hosts, causing cryptosporidiosis, and are the second most common cause of diarrhea after rotavirus in children [1]. The host’s immune state plays a key role in determining susceptibility to infection as well as the severity this disease. In healthy people, cryptosporidiosis causes self-limiting acute diarrhea, but immunocompromised individuals, such as children and people with acquired immunodeficiency syndrome (AIDS), can develop severe watery diarrhea, possibly leading to death [2]. *Cryptosporidium parvum* is the cause of the majority of human cryptosporidial infections [3]. Significantly, the development of vaccines or effective therapeutics to control cryptosporidiosis remains challenging. Therefore, more advanced tools coupled with large-scale data analysis are required for deep investigations of these causes them.

In recent years, accumulating evidence has shown that non-coding RNAs (ncRNAs) have a series of crucial regulatory functions both during transcription and post-transcription, and participate in many biological processes [4]. Initially, long ncRNAs (lncRNAs) were defined as a type of ncRNA that is longer than 200 nucleotides (nts) [5]. Recent evidence suggests that lncRNAs play a significant role in regulating epithelial defense against *C. parvum* infection. For example, lncRNA NR_045064 promotes defense gene transcription and facilitates intestinal epithelial cell responses against *Cryptosporidium* infection [6], and lncRNA NR_033736 regulates type I interferon-mediated gene transcription and modulates intestinal epithelial anti-*Cryptosporidium* defense [7].

MicroRNAs (miRNAs) are small, endogenous ncRNAs ranging from 19 to 24 nts that regulate post-transcriptional events by cleaving their target messenger RNAs (mRNAs) or by preventing translation [8]. At least seven host miRNAs are thought to be active in the innate immune defense against *Cryptosporidium* infection: *let-7i*, miR-98, miR-503, miR-424, miR-513, miR-221 and miR-27b [9–16]. Circular RNA (circRNA) is a type of closed-loop structure in the RNA molecule that is formed by special selective splicing of more than one exon [17]. Research has shown that the circRNA ciRS-7 has a miR-1270 binding site that interacts competitively with miRNAs, functioning as a miRNA sponge to regulate the transcription of miRNA-targeted genes and promote *C. parvum* propagation by affecting the nuclear factor kappa-B (NF-κB) pathway [18].

The miRNAs, lncRNAs and circRNAs induced by *C. parvum* infection are involved in regulating the biological function of the hosts [19–21]. In a previous study, we analyzed the miRNAs expressed in human ileocecal adenocarcinoma cells (HCT-8) cells during the early phase of *C. parvum* infection by using microarray analysis, and produced a limited set of data on differentially expressed genes [19]. In the present study, we investigated and analyzed the whole transcriptome sequence of HCT-8 cells infected with *C. parvum*. These results will provide a theoretical basis for further exploring the regulatory mechanisms of host ncRNAs during *C. parvum* infection.

## Methods

### Cell culture, parasites and in vitro infection model

Human ileocecal adenocarcinoma cells (HCT-8; American Type Culture Collection, Manassas, VA, USA) were cultured and maintained in RPMI 1640 medium supplemented with 10% fetal bovine serum (FBS), 4 mmol/l l-glutamine, 100 U/ml penicillin and 100 U/ml streptomycin at 37 °C in a humidified 5% CO_2_ incubator. *Cryptosporium parvum* subtype IId oocysts were initially obtained in China from neonatal dairy calves with clinical signs of diarrhea, and subsequently stored in a 2.5% K_2_Cr_2_O_7_ solution at 4 °C after purification. The *C. parvum* oocysts were purified using Sheather’s sugar flotation technique and cesium chloride density gradient centrifugation, sterilized with 10% sodium hypochlorite and stored at 4 °C until use. As previously described, immediately prior to use, oocysts were placed in 0.25% trypsin and 0.75% sodium taurocholate for 1 h with mixing every 5 min, followed by incubation at room temperature for 30 min and three washes in phosphate-buffered saline before being resuspended [22, 23]. HCT-8 cells (1.25 × 10^6^) were seeded into each well of a fresh 6-well plate with RPMI 1640 medium and supplemented with 10% FBS in 5% CO_2_ at 37 °C. The oocysts (1 × 10^7^/well) were incubated with HCT-8 cells and cultured for 3 h and 12 h (or until 80% confluence) with the ratio of oocysts:cells = 5:1. Cells without parasites were used as the controls.

### Sample collection, RNA extraction and quality monitoring

The cell samples were collected from both infected cells (named the infection group) and uninfected cells (named the control group) at 3 h post infection (hpi) and 12 hpi with sporozoites. Total RNA was extracted from three independent experiments using TRIpure Reagent (Aidlab, Beijing, China) following the manufacturer’s instructions. The concentration and quality of RNA were measured with the NanoDrop One Spectrophotometer (Thermo Fisher Scientific, Waltham, MA, USA) and the Agilent 2100 Bioanalyzer (Agilent Technologies, Santa Clara, CA, USA). Purified RNA was stored at –80 °C.

### RNA library construction and RNA sequencing

For the construction of the lncRNA library (a chain-specific library for removal of ribosomal RNA [rRNA]), the MGIEasy rRNA Removal Kit (MGI Tech Co., Ltd., Shenzhen, China) was used to remove the rRNA. The rRNA-depleted RNA was fragmented at a specific temperature and ion environment after purification. The complementary DNA (cDNA) strand was then synthesized using random primers and reverse transcriptase in the MGIEasy RNA Directional Library Preparation Kit (MGI Tech Co., Ltd.), and the double-stranded cDNA was then synthesized using DNA polymerase I and RNaseH. The double-stranded cDNA product was attached to an “A” base, followed by adaptor ligation. Finally, the link product was amplified, the PCR product was thermally denatured into a single strand and the single-strand DNA was cycled with a bridge primer to obtain the single-strand circular DNA library, which was sequenced on a DNBSEQ platform.

The construction of the small RNA library included the following main steps: (i) purification of the total RNA by polyacrylamide gel electrophoresis (PAGE) to separate RNA fragments of 18–30 nts; (ii) ligation of the 5-adenylated and 3-blocked adaptor to the 3′ end of the small RNA fragment; (iii) addition of reverse transcription (RT) primers with unique molecular identifiers (UMI) to the system to hybridize with the 3′-splices attached to RNA; (iv) 5′-end adaptor ligation; (v) first-strand synthesis with the UMI-labeled primers; (vi) use of highly sensitive polymerase to amplify the cDNA with both 3′- and 5′-splices, to amplify the output; (vii) PAGE to separate PCR products in the range of 110–130 bp; (viii) quantitative and pooling cyclization of the library; (ix) quality tests conducted on the constructed library; and (x) sequencing of qualified libraries on DNBSEQ.

### Data processing

The lncRNA library was sequenced on the DNBSEQ high-throughput sequencing platform to produce a large amount of data, referred to here as the “raw data.” In order to ensure the accuracy of the information analysis, quality control of the original data is required to obtain a high-quality sequence (i.e. clean reads). To this end, we first used the filtering software SOAPnuke (https://github.com/BGI-flexlab/SOAPnuke) to filter out rRNA, low-quality sequences, adaptor contamination and reads with too many N bases. The filtered “clean reads” were saved in the FASTQ format [24]. Clean reads were then aligned to the reference genome and transcriptome, respectively, using HISAT [25].

Similarly, the small RNA library sequence obtained by sequencing is processed by adaptor removal, low-quality data removal and de-contamination to complete the data processing and obtain a credible target sequence for backup analysis, and to allow analyses of sequence length distribution statistics and public sequence statistics among samples. The filtered data are referred to here as “clean tags” and are still stored in FASTQ format. The reads obtained from the sequencing were aligned with the reference genome using Bowtie2 [26].

### Analysis of differentially expressed genes

The DESeq [27] package within R (3.2.0) was used to analyze the inter-sample differential expression of predicted lncRNAs, miRNAs, circRNAs and mRNAs (dif-lncRNAs, dif-miRNAs, dif-circRNAs and dif-mRNAs, respectively). For each sample, the counts of lncRNAs, miRNAs, circRNAs and mRNAs were normalized to compute the fold change (FC), and the binomial distribution was used to test the significance of the differences between the *C. parvum* infection group and the control group. The |log_2_(FC)| ≥ 1 and *Q*-value  ≤ 0.05 were considered to be the thresholds for screening dif-lncRNAs, dif-miRNAs, dif-circRNAs,and dif-mRNAs.

### Quantitative real-time PCR validation

Quantitative real-time PCR (qRT-PCR) analyses were performed using the SYBR ®Green Realtime PCR Master Mix (TOYOBO Co., Ltd., Osaka, Japan) according to the manufacturer’s instructions. The internal references were the glyceraldehyde 3-phosphate dehydrogenase gene (*GAPDH*) for circRNA, lncRNA and mRNA) and the U6 gene (*U6*) for miRNA). The reaction conditions were: an initial incubation at 95 °C for 30 s; followed by 40 cycles of 5 s (denaturation) at 95 °C, 34 s (annealing) at 55 °C and 15 s (extension) at 72 °C. The average cycle threshold value (Ct value) was used to calculate the relative expression of dif-lncRNA, dif-miRNA, dif-circRNA and dif-mRNA using the comparative 2^–△△Ct^ method. All experiments were carried out in triplicate. The primer sequences are presented in Additional file 1: Table S1.

### Target prediction of dif-lncRNAs, dif-miRNAs and dif-circRNAs

The target genes of dif-lncRNAs include two regulatory modes, namely the *cis-* and *trans-*acting modes. Potential target mRNAs within 100 kb upstream and downstream of the dif-lncRNAs were searched for the prediction of *cis-* targets, while the potential *trans-* target genes were selected with complementary or similar sequences to lncRNA by RNAplex [28] software, with the criterion of direct complementary base pairs  ≥ 10 and free energy  ≤  − 100 kcal/mol. The speculated target genes of dif-miRNAs were also predicated using miRanda [29] and RNAhybrid [30] software. In addition, the binding sites of dif-circRNAs at miRNAs were predicted using miRanda software.

### Gene Ontology annotation and Kyoto Encyclopedia of Genes and Genomes enrichment analysis

To better understand the biological functions and potential mechanisms of ncRNAs and mRNAs in the mechanism of *C. parvum* infection, we applied Gene Ontology (GO) and Kyoto Encyclopedia of Genes and Genomes (KEGG) enrichment analyses to these dif-mRNAs, sponging miRNA target genes of dif-circRNA, predicted target genes of dif-miRNAs and predicted target genes of dif-lncRNAs. Briefly, the GO analysis (http://www.geneontology.org) consists of three components: biological processes (BP), cellular component (CC) and molecular function (MF). KEGG analysis was carried out to investigate the potential significant pathways (http://www.genome.jp/kegg/). The enrichment analysis of GO terms and KEGG pathways with a significance threshold were considered to be significant at *P*-value  < 0.05.

### The construction of the competing endogenous RNA regulatory network

To reveal the interactions among the dif-mRNAs, dif-miRNAs, dif-lncRNAs and dif-circRNAs, we constructed a competing endogenous RNA (ceRNA) regulatory network using circRNA/lncRNA-miRNA-mRNA based on the ceRNA hypothesis. The dif-miRNA-dif-mRNA, dif-miRNA-dif-lncRNA and dif-miRNA-dif-circRNA pairs were predicted. Lastly, these two complex networks of circRNA-miRNA-mRNA and lncRNA-miRNA-mRNA were integrated, and we focused on screening miRNAs that can regulate circRNA, lncRNA and mRNA. The target relationships of mRNA, lncRNA and circRNA regulated by these miRNAs were further obtained. The open source software platform Cytoscape v3.5.1 was applied to build a ceRNA network.

### Statistical analysis

Statistical analysis was performed using GraphPad Prism ver. 8.0.2 (GraphPad Software, Inc., San Diego, CA, USA). The expression level of each gene was represented as a FC by the 2^–△△Ct^ method. Student’s t-test was used to analyze the differences between the two groups. All data are expressed as the mean ± standard deviation. All experiments were performed on no fewer than three biological replicates. Significance was defined as a *P*-value  < 0.05.

## Results

### Identification and analysis of differentially expressed ncRNAs and mRNAs

A total of 1338.42 M raw reads and 1328.14 M clean reads were obtained from 12 cell samples in the construction of the lncRNA library, with an average of 11.07 Gb clean reads per sample. The mean Q30 score was 93.96% (i.e. probability of a correct base call was 93.96%), demonstrating that the good quality of the RNA-sequencing (RNA-seq) data. Similarly, an average yield of 32.67 M reads per sample was obtained in the small RNA library, and the average alignment ratio of the sample comparison genome was 88.67%, showing the good quality of the RNA-seq data. Additionally, a total of 23,912 lncRNA transcripts, 17,839 mRNAs, 51,358 circRNAs and 2076 miRNAs were detected.

**Fig. 1:**
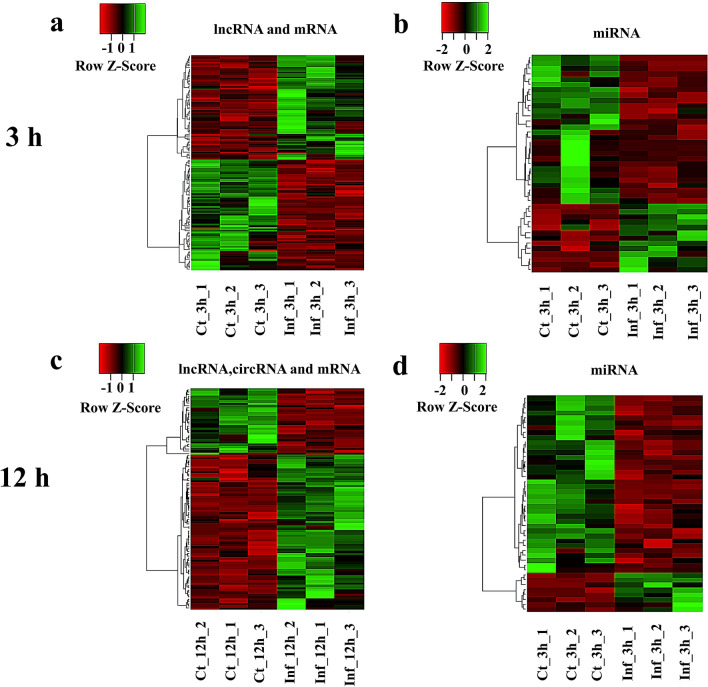
Heatmaps of differentially expressed molecules in HCT-8 cells after *Cryptosporium parvum* infection.** a**,** b** Heatmaps of parts of dif-lncRNAs and dif-mRNA (**a**) and dif-miRNAs (**b**) in HCT-8 cells at 3 hours post infection (hpi).** c**,** d** Heatmaps of parts of dif-lncRNAs, dif-circRNA and dif-mRNA (**c**) and dif-miRNAs (**d**) in HCT-8 cells at 12 hpi. The horizontal axis indicates samples of *C. parvum* infected (Inf) cells and control (Ct) cells. Red indicates upregulation, and green indicates downregulation. dif-lncRNA, Differentially expressed long non-coding RNA, dif-circRNA, differentially expressed circular RNA; dif-mRNA, differentially expressed messenger RNA; dif-miRNA, differentially expressed microRNA; HCT-8, human ileocecal adenocarcinoma cells

According to the screening criteria, 115 (61 upregulated, 54 downregulated) mRNAs, 393 (218 upregulated, 175 downregulated) lncRNAs and 69 (21 upregulated, 48 downregulated) miRNAs were differentially expressed in HCT-8 cells at 3 hpi. In addition, 117 (78 upregulated and 39 downregulated) mRNAs, 450 (284 upregulated and 166 downregulated) lncRNAs, 129 (48 upregulated and 81 downregulated) miRNAs and one upregulated circRNA were differentially expressed in HCT-8 cells at 12 hpi (Additional file 2: Table S2). Additionally, 94 dif-lncRNAs, 24 dif-miRNAs and 22 dif-mRNAs were commonly identified at both time points post infection, suggesting they might play essential roles in the pathogenesis of *C. parvum* infection. Parts of the clustering heatmaps of dif-mRNA, dif-miRNA, dif-lncRNA and dif-circRNA are shown in Fig. 1, which shows that the cell samples could be significantly separated from the control samples, indicating that the results of the analysis of differential expression were reliable.

### qRT-PCR validation analysis

**Fig. 2:**
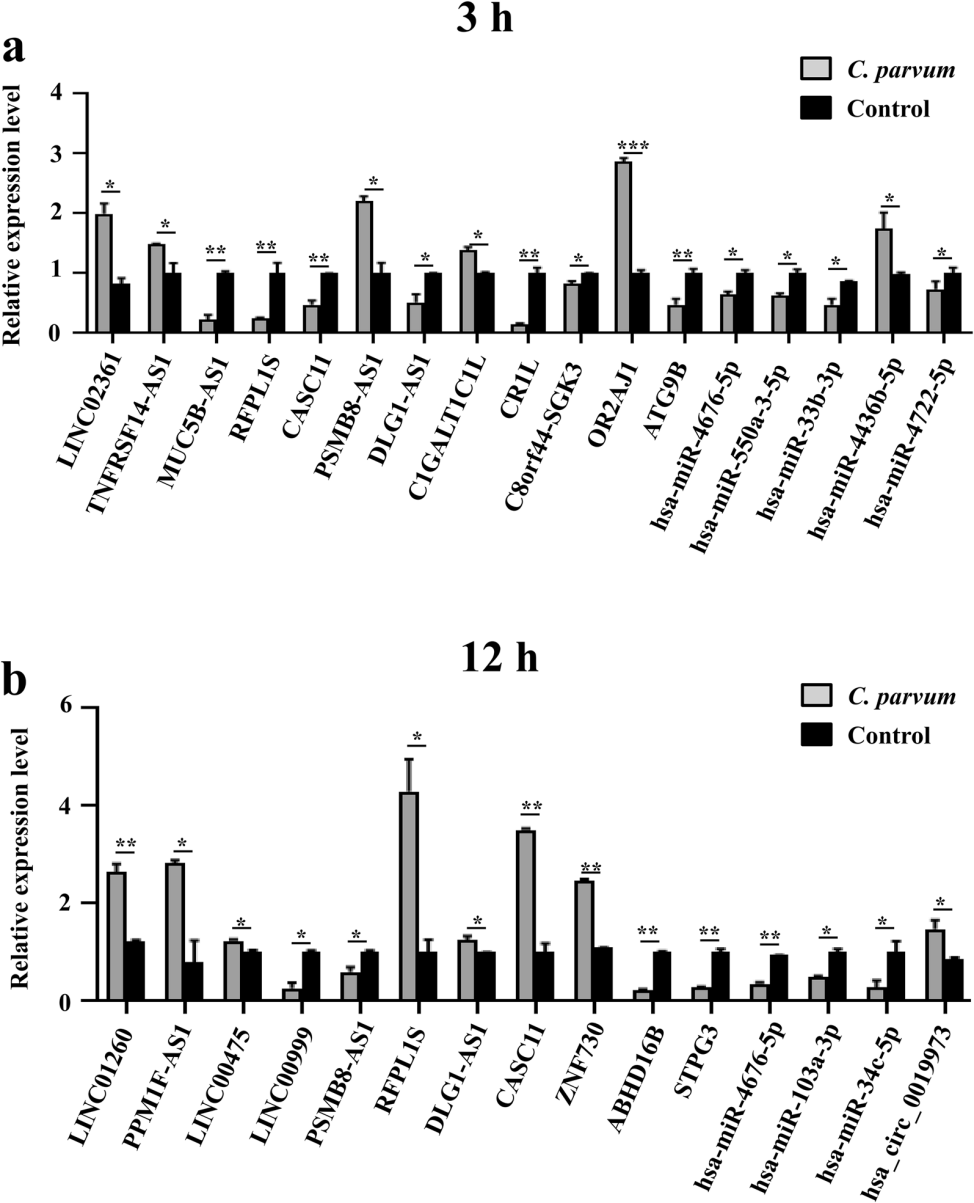
Validation of the differentially expressed genes. Quantitative real-time PCR confirmation of the lncRNAs, miRNAs and mRNAs differentially expressed at 3 hpi (**a**) and the lncRNAs, miRNAs, circRNAs and mRNAs differentially expressed at 12 hpi (**b**). Three biological repeats were included for each gene. The names of genes and their relative expression are represented by the horizontal and vertical axes, respectively. Asterisks indicate a significant difference in expression at *P < 0.05, **P < 0.01, ***P < 0.001

To validate the accuracy and reliability of the RNA-seq data, qRT-PCRs were performed to determine the levels of expression of 11 lncRNAs (5 upregulated and 2 downregulated), eight mRNAs (3 upregulated and 5 downregulated), one upregulated circRNA and seven miRNAs (1 upregulated and 7 downregulated) that were randomly selected from the RNA-seq data. As shown in Fig. 2, the results from sequencing data were in agreement with those from qRT-PCR in terms of the levels of expression of the validated differentially expressed genes.

### Biological function analysis

**Fig. 3:**
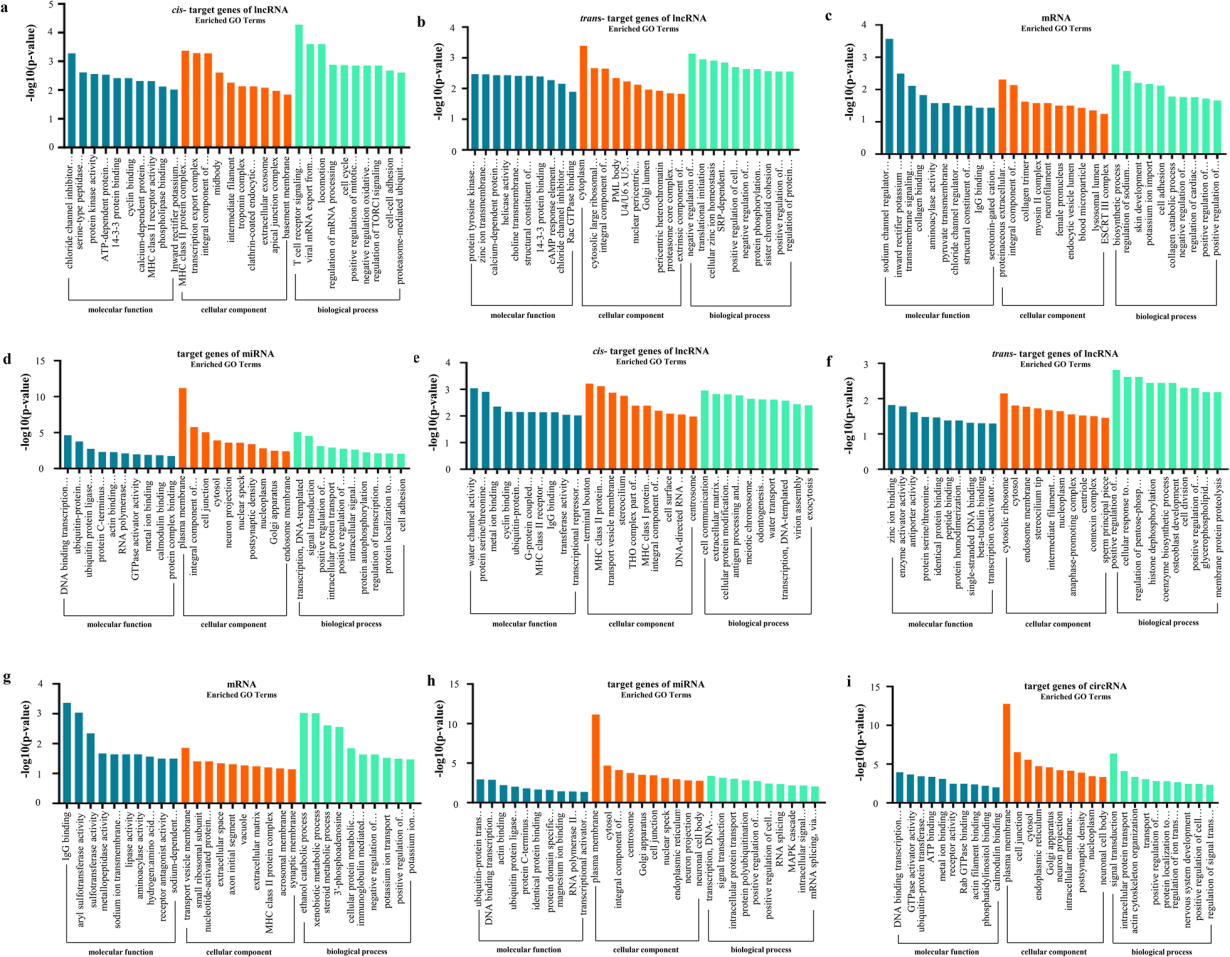
GO annotation.** a**,** b** Bar charts of GO annotation analysis for the targets of lncRNAs, dif-mRNAs (**a**), and targets of miRNAs (**b**) in HCT-8 cells at 3 hpi.** e**–**i** Bar charts of GO annotation analysis for the target of lncRNAs, dif-mRNAs, target of miRNAs, and target of circRNA in HCT-8 cells at 12 hpi. The ontology covered three domains: biological processes, cellular components, and molecular functions. GO, Gene Ontology

**Fig. 4:**
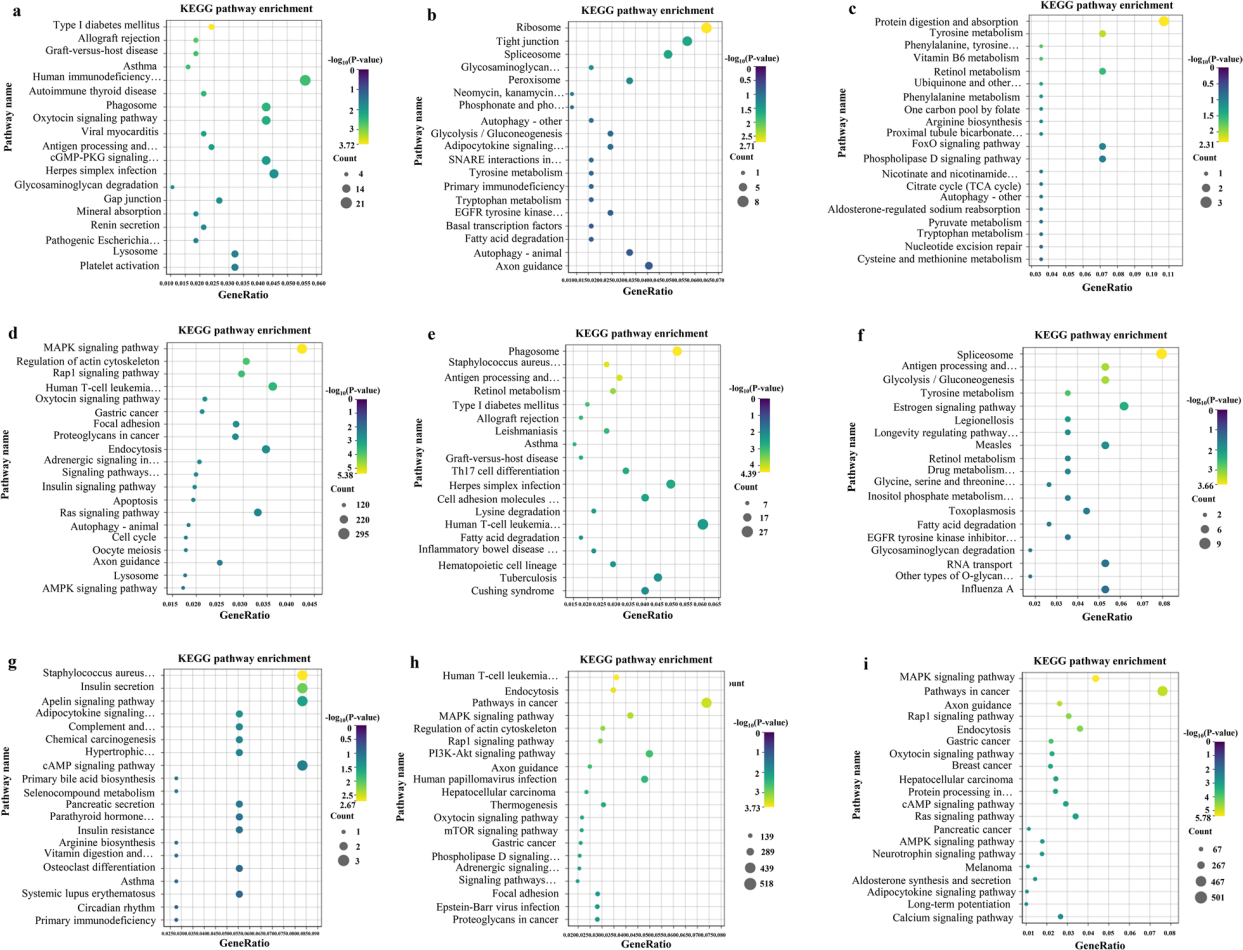
Analysis of the KEGG pathway. **a**–**d**The top 20 pathways enriched by genes regulated by differentially expressed lncRNAs, mRNAs, miRNAs in HCT-8 cells at 3 hpi. **e**–**i** The top 20 pathways enriched by genes regulated by differentially expressed lncRNAs, mRNAs, miRNAs, circRNA in HCT-8 cells at 12 hpi. The vertical axis represents the pathway name and the horizontal axis represents the gene ratio. The size of the filled circle represents the number of differentially expressed genes in the pathway, and the color of the filled circle corresponds to the different -log_10_ (*P*-value) ranges. KEGG, Kyoto Encyclopedia of Genes and Genomes

A total of 2900 *cis*-target and *trans*-target genes were screened for 843 dif-lncRNA transcripts (Additional file 3: Table S3). The results of GO annotation showed that 310 GO terms were significantly enriched at 3 hpi, of which 195 belonged to the BP category (e.g. T-cell receptor signaling pathway, cell cycle, proteasome-mediated ubiquitin-dependent protein catabolic process, protein phosphorylation), 59 belonged to the CC category (e.g. MHC class II protein complex, intermediate filament, extracellular exosome, cytoplasm, cytosolic large ribosomal subunit) and 89 belonged to the MF category (e.g. serine-type peptidase activity, protein kinase activity, ATP-dependent protein binding). A total of 238 GO terms were also significantly enriched at 12 hpi, of which 148 belonged to the BP category (e.g. antigen processing and presentation of exogenous peptide antigen via MHC class II, transcription, DNA-template and cellular protein modification process), 44 belonged to the CC category (e.g. cell surface, terminal bouton and transport vesicle membrane) and 46 belonged to the MF category (e.g. metal ion binding, IgG binding, ubiquitin-protein transferase activity and zinc ion binding) (Additional file 4: Table S4). The top 30 significantly enriched GO terms are shown in Fig. 3a, b, e, f. At 3 hpi, 38 pathways were significantly enriched; for example the tight junction protein. At 12 hpi, 52 pathways were significantly enriched; for example the cell adhesion molecules (CAMs) pathway (Additional file 4: Table S4). The top 20 most representative pathways in each group are shown in Fig. 4a, b, e, f.

The functional classification and enrichment analysis of dif-mRNAs showed that 46 dif-mRNAs were significantly enriched in 80 GO terms at 3 hpi. Of these, 54 belonged to the BP category (e.g. cell adhesion, biosynthetic process, regulation of sodium ion transmembrane transporter activity), nine belonged to the CC category (e.g. proteinaceous extracellular matrix, integral component of plasma membrane) and 17 belonged to the MF category (e.g. sodium channel regulator activity, transmembrane signaling receptor activity, IgG binding). At 12 hpi, 41 dif-mRNAs were significantly enriched in 80 GO terms. Of these, 51 belonged to the BP category (e.g. cellular protein metabolic process and xenobiotic metabolic process), five belonged to the CC category (e.g. transport vesicle membrane and extracellular space) and 24 belonged to the MF category (e.g., IgG binding and sodium ion transmembrane transporter activity) (Additional file 5: Table S5). The top 30 significantly enriched GO terms are shown in Fig. 3c, g. Moreover, KEGG enrichment analysis at 3 hpi showed that six dif-mRNAs were significantly enriched in six signaling pathways (e.g. protein digestion and absorption, tyrosine and retinol metabolism). At 12 hpi, nine dif-mRNAs were significantly enriched in four signaling pathways (e.g. apelin signaling pathway and adipocytokine signaling pathway) (Additional file 5: Table S5). The top 20 most representative pathways in each group are shown in Fig. 4c, g.

The GO annotation results for predicted target genes of dif-miRNAs showed that 81 GO terms were significantly enriched at 3 hpi. Of these, 34 belonged to the BP category (e.g. transcription, DNA-templated, signal transduction, positive regulation of transcription from RNA polymerase II promoter, positive regulation of cell proliferation and cell adhesion), 31 belonged to the CC category (e.g. cytosol, plasma membrane, integral component of plasma membrane, nucleoplasm and Golgi apparatus) and 16 belonged to the MF category (e.g. DNA binding transcription factor activity, GTPase activator activity, actin binding and metal ion binding). Additionally, 66 GO terms were significantly enriched at 12 hpi. Of these, 24 belonged to the BP category (e.g. transcription, DNA-templated and signal transduction), 30 belonged to the CC category (e.g. endoplasmic reticulum and plasma membrane) and 12 belonged to the MF category (e.g. identical protein binding and DNA binding transcription factor activity) (Additional file 6: Table S6). The top 30 significantly enriched GO terms are shown in Fig. 3d, h. At 3 hpi, 57 pathways were significantly enriched; for example, the mitogen-activated protein kinase (MAPK) signaling pathway and apoptosis. At 12 hpi, 53 pathways were significantly enriched; for example the MAPK signaling pathway and phosphatidylinositol 3 kinase (PI3K-Akt) signaling pathway (Additional file 6: Table S6). The top 20 most representative pathways in each group are shown in Fig. 4d, h.

The 34 potential target miRNAs of the significantly upregulated circRNA hsa_circ_0019973 was predicted based on sequence complementarity, using bioinformatics analysis, and the potential functions of hsa_circ_0019973 were annotated using GO and KEGG enrichment analysis of 18,334 sponging miRNA target genes. The GO annotation results of 18,334 sponging miRNA target genes showed that 148 GO terms were significantly enriched at 12 hpi. Of these, 55 belonged to the BP category (e.g. signal transduction, actin cytoskeleton organization, positive regulation of transcription from RNA polymerase II promoter and positive regulation of cell proliferation), 63 belonged to the CC category (e.g. plasma membrane, cytosol, Golgi apparatus and nucleoplasm) and 30 belonged to the MF category (e.g. DNA binding transcription factor activity, GTPase activator activity, ATP binding, metal ion binding and calmodulin binding) (Additional file 7: Table S7). The top 30 significantly enriched GO terms are shown in Fig. 3i. At 12 hpi, 94 pathways were significantly enriched; for example, the MAPK signaling pathway and AMPK signaling pathway (Additional file 7: Table S7). The top 20 most representative pathways in each group are shown in Fig. 4i.

### Construction and analyses of ceRNA networks

**Fig. 5:**
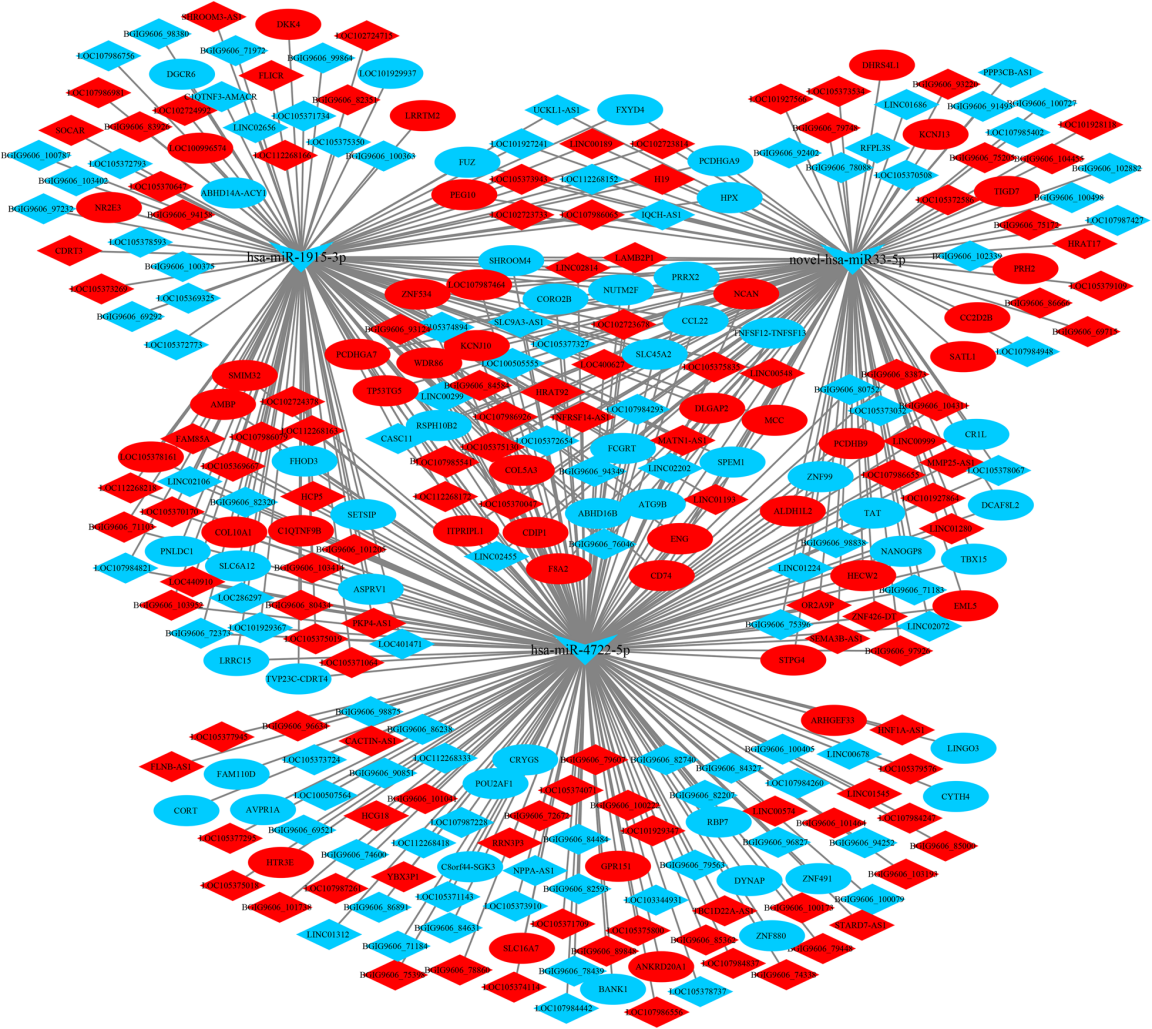
The lncRNA-miRNA-mRNA network at 3 hpi. Red represents the upregulated genes, blue indicates downregulated genes. The ellipse, V and diamond shapes in this diagram represent mRNA, miRNA and lncRNA, respectively

**Fig. 6:**
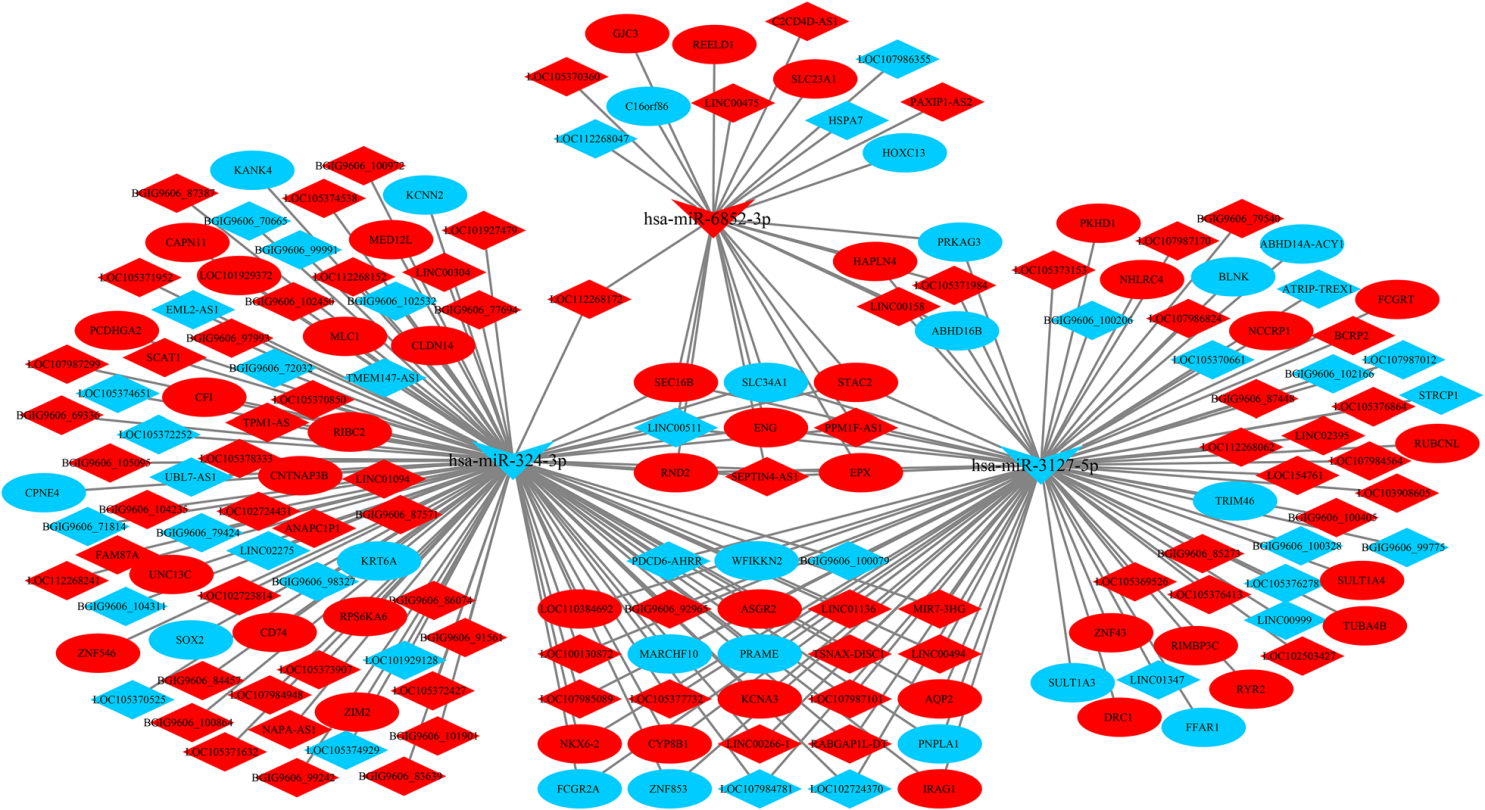
The lncRNA-miRNA-mRNA network at 12 hpi. Red represents the upregulated genes, blue indicates downregulated genes. The ellipse, V and diamond shapes in this diagram represent mRNA, miRNA and lncRNA, respectively

Both lncRNA and circRNA are known to sponge miRNAs to prevent their interactions with target mRNAs, thus exhibiting competitive endogenous RNA (ceRNA) activity [18, 31]. We constructed lncRNA-miRNA-mRNA networks based on the relationship of the target of the lncRNA and miRNA, and the target of the mRNA and miRNA. The target lncRNAs of miRNAs were predicated using miRanda software [29], and the target mRNAs of the miRNAs were predicated by the miRanda and RNAhybrid databases. In total, we obtained 302 lncRNA-miRNA-mRNA interactions at 3 hpi with *C. parvum* (Fig. 5), including 213 lncRNAs, 86 mRNAs and three miRNAs (hsa-miR-4722-5p; hsa-miR-1915-3p; novel-hsa-miR33-5p). Additionally, we obtained 175 lncRNA-miRNA-mRNA interactions after 12 hpi with *C. parvum*, including 110 lncRNAs, 62 mRNAs and three miRNAs (hsa-miR-324-3p; hsa-miR-6852-3p; hsa-miR-3127-5p) (Fig. 6).

**Fig. 7:**
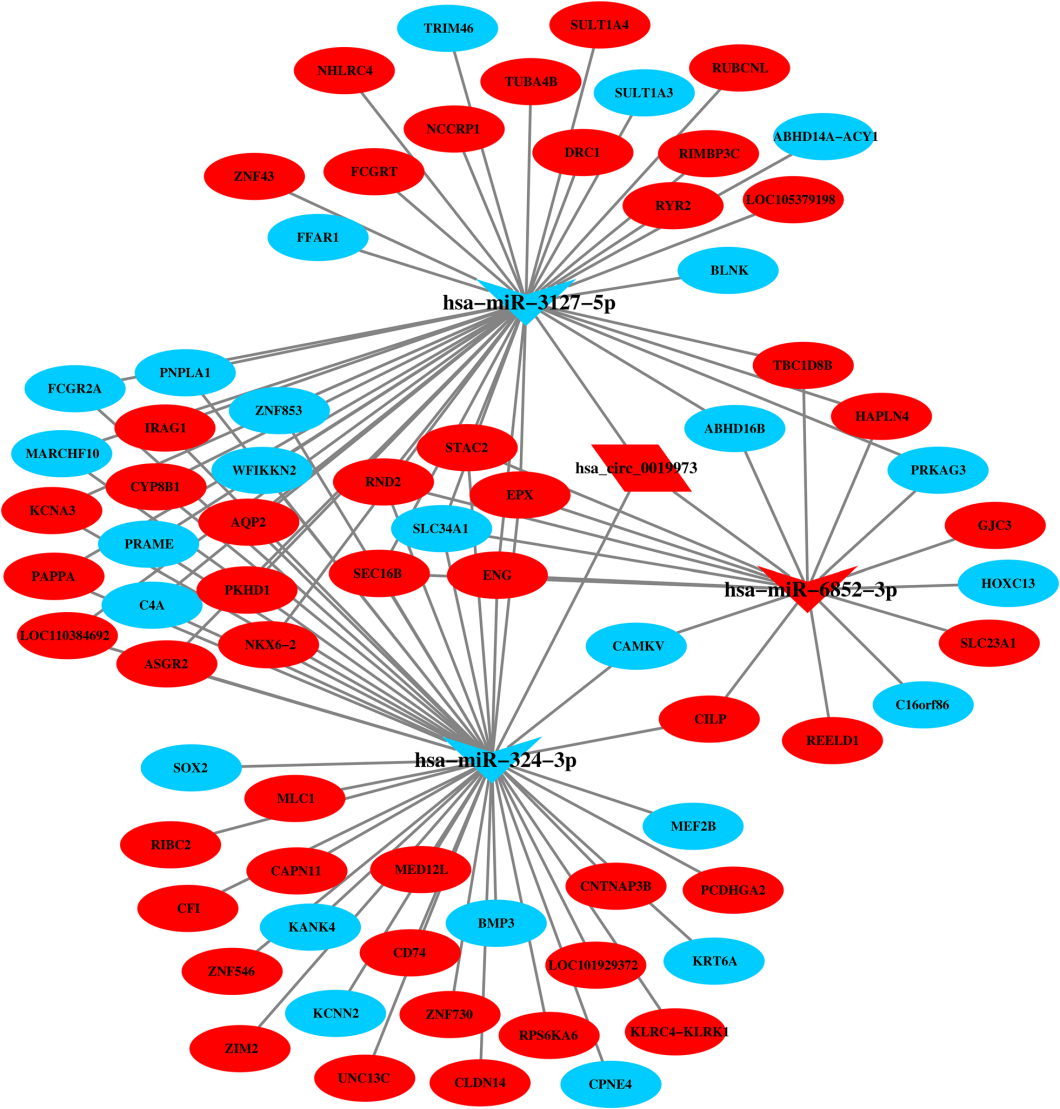
The circRNA-miRNA-mRNA network at 12 hpi. Red represents the upregulated genes, blue indicates downregulated genes. The ellipse, V and parallelogram shapes in this diagram represent mRNA, miRNA and circRNA, respectively

Similarly, dif-circRNA and mRNA regulated by the same miRNAs were screened on the basis of the regulatory relationship of dif-miRNA-dif-mRNA and dif-miRNA-dif-circRNA. Eventually, we found 76 interactive relationships of circRNA-miRNA-mRNA after 12 hpi with *C. parvum* (Fig. 7), of which there was one circRNA, 72 mRNAs and three miRNAs (hsa-miR-3127-5p; hsa-miR-6852-3p; hsa-miR-324-3p).

**Fig. 8:**
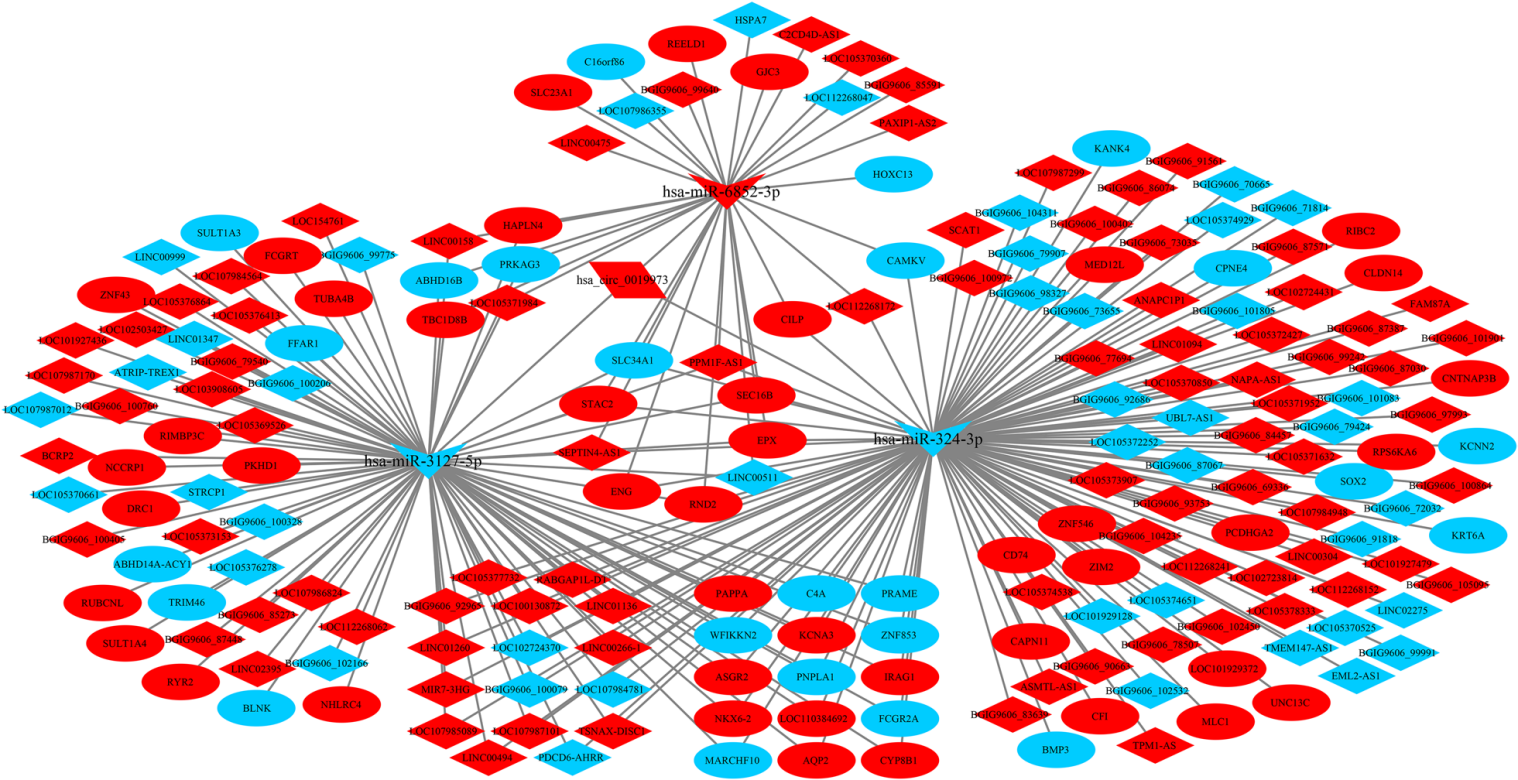
The competing endogenous RNA (ceRNA) network at 12 hpi. Red represents the upregulated genes, blue indicates downregulated genes. The ellipse, V, diamond and parallelogram shapes in this diagram represent mRNA, miRNA, lncRNA and circRNA, respectively

Based on the lncRNA-miRNA-mRNA and circRNA-miRNA-mRNA networks after 12 hpi with *C. parvum*, we further screened the differentially expressed circRNAs, lncRNAs and mRNAs that were regulated by the same miRNA. Ultimately, 201 interaction pairs were obtained (Fig. 8), including one circRNA, 129 lncRNAs, 68 mRNAs and three miRNAs (hsa-miR-324-3p; hsa-miR-6852-3p; hsa-miR-3127-5p).

## Discussion

RNA-Seq is a recently developed approach to transcriptome profiling that provides more data and identifies novel transcripts [32]. In this study, a total of 1042 ncRNAs and 232 mRNAs of HCT-8 cells were differentially expressed at 3 hpi and 12 hpi, which is clearly higher than the number obtained in our previous study by microarray analysis [19]. Differently expressed ncRNAs and mRNAs at different time points might represent functional differences in these RNAs during the development of HCT-8 cells infected by *C. parvum* [21].

Previous studies have demonstrated that dysregulation of mRNAs, such as FCGR2A, PNPLA1, SOX2, MLC1, EPX, CD74 and TAT, plays an important role in the process of Apicomplexa infection [33–40]. FCGR2A, a member of a family of immunoglobulin Fc receptor genes, was downregulated at 12 hpi and upregulated at 24 hpi in our study, while it has been shown to belong to the IgG binding, Fc-gamma receptor signaling pathway involved in phagocytosis, plasma membrane and extracellular exosome at both 12 hpi and 24 hpi [21], indicating that this gene might be relevant to immune response associated with *C. parvum* infection. Additionally, KEGG enrichment analysis has shown that these dif-mRNAs are significantly enriched in nutritional absorption, metabolic processes and metabolism-related pathways. However the role of each dif-mRNA needs to be explored in the future. A number of earlier studies have confirmed that *C. parvum* infection is closely associated with these pathways in biliary epithelial cells [41–44].

MiRNAs have been confirmed to regulate the innate immune response to *C. parvum* infection [9–16]. The GO and KEGG enrichment analyses in the present study showed that the target genes of dif-miRNAs were significantly enriched in the apoptosis and apoptosis-related pathways, which is consistent with results reported from a microarray sequencing study [19]. Previous studies have shown that *C. parvum* infection inhibits intestinal epithelial cell apoptosis during the early stage of infection [45]. Nevertheless, studies of the mechanisms regulating host apoptosis are few, especially as related to miRNAs. The B7-H1 gene, targeted by miR-513, contributes to the regulation of the apoptosis of human cholangiocytes induced by *C. parvum* [14]. In addition, hsa-miR-324-3p and hsa-miR-3127-5p are the key miRNAs in the complex ceRNA network that regulates the differentially expressed lncRNAs, circRNAs and mRNAs, representing a key role in the regulatory network. The ceRNA network involved in cell apoptosis has been demonstrated in other studies. For example, the lncRNA H1FX-AS1 has been shown to induce apoptosis by sponging hsa-miR-324-3p to upregulate the level of DACT1 expression in cervical cancer [46]. The circRNA Rno_circ_0005139 was found to influence cell proliferation and apoptosis by acting as a miR-324-3p sponge, thereby downregulating Wnt5a in a rat anorectal malformation [47]. The circRNA circPAPPA regulates the apoptosis of trophoblast cells through the miR-3127-5p/HOXA7 axis [48].

The dif-lncRNAs identified in this study were mainly involved in pathways related to the infection and pathogenicity of *Cryptosporium*. Studies have suggested that *Crytosporium andersoni* is able disrupt the integrity of the tight junction zonula-occludens-1 (ZO-1) protein of bovine epithelial cells [49]. *Crytosporium parvum* has been closely associated with CAMs in epithelial cells, disrupting intestinal epithelial barrier function by altering the expression of key tight junction proteins [50, 51]. Additionally, our results showed that several lncRNAs, such as HCG18, MATN1-AS1 and NEBL-AS1, were upregulated at 3 hpi, and that the lncRNAs NPPA-AS1 and MIR7-3HG were also upregulated at 12 hpi, consistent with previous studies using HCT-8 cells infected with *C. parvum* at 24 hpi [21]. These findings indicate these host lncRNAs might play an important role in the infection of *C. parvum*. Furthermore, one lncRNA, LOC100132686, was significantly downregulated, and its potential target interleukin 18 has been identified as an important cytokine in the defenses against *Cryptosporidium* infection [52, 53], suggesting that potential target genes of dysregulated lncRNAs might play important roles in regulation of the interaction between host cells and *C. parvum*.

CircRNAs may have a variety of potential biological functions, such as miRNA target decoys, RNA binding protein sponges and transcriptional regulators [54–56]. In the present study, only one upregulated circRNA, has_circ_0019973, may indirectly regulate 18,334 mRNAs through sponging 34 miRNAs, indicating that one circRNA contained more than one miRNA binding sites. In addition, the functions analysis of this circRNA revealed that the MAPK signaling pathway was significantly enriched by sponging miRNA target genes. These data on the roles of dif-circRNA need to be explored in the future. Nevertheless, a previous study has suggested that miR-1270 activates the NF-κB signaling pathway in response to *C. parvum* infection, and its interacting circRNA ciRS-7 was also found to be differentially expressed [18]. In the present study, based on the constructed circRNA-miRNA-mRNA regulatory network, we observed that hsa_circ_0019973 was able to interact with RPS6KA6, which participates in the MAPK signaling pathway, through competitive binding with hsa-miR-324-3p. Further study is required to reveal the interaction relationships of hsa_circ_0019973-hsa-miR-324-3p-RPS6KA6 in *C. parvum* infection.

## Conclusions

In conclusion, the whole transcriptome profiles of HCT-8 cells were analyzed after *C. parvum* infection using RNA-seq. A total of 232 mRNAs and 1042 differently expressed ncRNAs were identified at 3 hpi and 12 hpi, with 27 RNAs confirmed by qRT–PCR. The functional prediction using GO and KEGG pathway analyses implicated significant roles of these differently expressed RNAs during the course of *C. parvum* infection. According to the ceRNA regulatory network, a single lncRNA or circRNA can be connected with numerous miRNAs, which subsequently coregulate additional mRNAs. In addition, the constructed ceRNA regulatory networks suggest that the downregulated hsa-miR-324-3p and hsa-miR-3127-5p may be the key molecules that regulate circRNAs, lncRNAs and mRNAs. These findings provide novel insights into the responses of human intestinal epithelial cells to *C. parvum* infection.

## Supplementary Information

**Additional file 1:** **Table S1.** Primers designed for qRT-PCR validation of candidate miRNAs, mRNAs, circRNAs and lncRNAs in HCT-8 cells at 3 h and 12 h post infection with *Cryptosporum parvum*.

**Additional file 2:** **Table S2. **The differentially expressed mRNAs, lncRNAs, miRNAs and circRNA at 3 hpi and 12 hpi.

**Additional file 3:** **Table S3.** The *cis*- and *trans*- target genes predicted for dif-lncRNAs at 3 hpi and 12 hpi.

**Additional file 4:** **Table S4.** The GO and KEGG enrichment analysis of *cis*‑ and *trans*- target genes for dif-lncRNAs at 3 hpi and 12 hpi.

**Additional file 5:** **Table S5. **The GO and KEGG enrichment analysis of dif-mRNAs at 3 hpi and 12 hpi.

**Additional file 6:** **Table S6. **The GO and KEGG enrichment analysis of target genes for dif-miRNAs at 3 hpi and 12 hpi.

**Additional file 7:** **Table S7. **The GO and KEGG enrichment analysis of sponging miRNA targets for the dif-circRNA at 12 hpi.

## Data Availability

The datasets supporting the findings of this article are included within the paper and its supplementary materials. The RNA-seq raw data described in the present study has been submitted to the NCBI Short Read Archive database (https://www.ncbi.nlm.nih.gov/sra) under the bio-project number PRJNA888239 and PRJNA883802.

## Abbreviations

BP: Biological process
CAMs: Cell adhesion molecules
CC: Cellular component
cDNA: Complementary DNA
ceRNA: Competing endogenous RNA
circRNAs: Circular RNAs
dif-RNAs: Differentially expressed RNAs
FC: Fold change
HCT-8 cells: Human ileocecal adenocarcinoma cells
GO: Gene Ontology
KEGG: Kyoto Encyclopedia of Genes and Genomes
lncRNAs: Long non-coding RNAs
mRNAs: messenger RNAs
miRNAs: MicroRNAs
MAPK: Mitogen-activated protein kinase
MF: Molecular function
ncRNAs: Non-coding RNAs
NF-κB: Nuclear factor kappa-light-chain-enhancer of activated B cells
PAGE: Polyacrylamide gel electrophoresis
PI3K: Phosphatidylinositol 3 kinase
qRT-PCR: Quantitative real-time PCR
RNA-seq: RNA-sequencing
rRNA: Ribosomal RNA
UMI: Unique molecular identifier
