# Supplementary material for: Whole transcriptome analysis of HCT-8 cells infected by Cryptosporidium parvum
Source: Parasit Vectors. 2022 Nov 24;15:441. doi: 10.1186/s13071-022-05565-4 (PMC9700907; doi:10.1186/s13071-022-05565-4)
Supplement: Supplementary file 1 — Additional file 1: Table S1. Primers designed for qRT-PCR validation of candidate miRNAs, mRNAs, circRNAs and lncRNAs in HCT-8 cells at 3 h and 12 h post infection with Cryptosporum parvum. [file 13071_2022_5565_MOESM1_ESM.docx]

**Additional file 1: Table S1.** Primers designed for qRT-PCR validation of candidate miRNAs, mRNAs, circRNAs and lncRNAs in HCT-8 cells at 3 h and 12 h post infection with *Cryptosporum parvum*.

| **Genes** | **Primers** |  |
| --- | --- | --- |
|  | **Forward** | **Reverse** |
| LINC02361 | CAGCTCCAGACCACCAGAG | GAGATGAGGAGGAACCGCAC |
| TNFRSF14-AS1 | GAGACAGGAGTTCCAACCGC | ACTTTGGTCTTCGTGTGGCT |
| MUC5B-AS1 | CTCTGTGAGGATCCAGTGGACG | AAAGAGCATAGAGTGCCGGG |
| LINC01260 | CTGCTTCCCATCCTCCATCC | GTCCTCTCTGGTGTGTGTGG |
| PPM1F-AS1 | CGACACTCCCGGCTTTAACT | TCCATCCCGGTAGATTCCTCA |
| LINC00475 | AGGGTTCACAAACGAGCCTG | GAATGCCCAGGCTCCTAACC |
| LINC00999 | TGACTCAAGCTACCACATGCT | TCCATCCCACGCCATTCTTC |
| PSMB8-AS1 | CGATGGGTTACAGTAAGAGCG | GTCGACAGTTGCTGGGTAGATG |
| RFPL1S | TCACAGTCCAGAATCCACGC | ACAGCCAACAACTTCCTCCTC |
| DLG1-AS1 | GAAGGCTCGGGCTGTCTGAA | AAGCAAAAGATGCAGCGGAG |
| CASC11 | GCTGCAGAAGGTCCGAAGAA | TTCACCACGTCCAGTTGCTT |
| C1GALT1C1L | TGGGAGCATTTCCTGGGTTTT | CGTTCCTGTTAGGTGGACGAA |
| CR1L | CGCACAAGTGAACCTCATGG | GACAGAATTCCTCAGCAAACACT |
| C8orf44-SGK3 | GCCTTGGGGCTGTTCTGTAT | AGACTCACTCCTGGCCTCAA |
| OR2AJ1 | CCCCAACAAGTGTGGTCTTCT | CATTGGAGTGTGGAGTCGGG |
| ATG9B | TGCCAACCAACCAAGTAACCATACC | CACTGGGCTGAGGGTAGGATGG |
| ZNF730 | AAAGCCTTTAGCCGTATCTCACACC | AAGGGTTGAGGACTGGTTGAAAGC |
| ABHD16B | TGGTCTACGGCTGGTCTGTTGG | CAACTGTGGGGCATGACCTTCAG |
| STPG3 | GATGAGACACAGCCCCCAAA | CTCAGGGTCTGGGTGTAGGT |
| hsa-miR-4676-5p | AGTATCCGAGCCAGTGGTGAG | AGTGCAGGGTCCGAGGTATT |
| hsa -miR-550a-3-5p | AGAGACGAGTGCCTGAGGGA | AGTGCAGGGTCCGAGGTATT |
| hsa -miR-33b-3p | GCAGTGCCTCGGCAGTG | AGTGCAGGGTCCGAGGTATT |
| hsa -miR-4436b-5p | CGGTCCACTTCTGCCTGC | AGTGCAGGGTCCGAGGTATT |
| hsa -miR-4722-5p | GGCAGGAGGGCTGTGCC | AGTGCAGGGTCCGAGGTATT |
| hsa -miR-103a-3p | GCGAGCAGCATTGTACAGGG | AGTGCAGGGTCCGAGGTATT |
| hsa -miR-34c-5p | CGCGAGGCAGTGTAGTTAGCT | AGTGCAGGGTCCGAGGTATT |
| hsa_circ_0019973 | GGGGCAGGAGTCAGCAATAAAG | GTAGATGCGGATCGAGCCCT |
| *U6* | GCTTCGGCAGCACATATACAAAAT | CGCTTCACGAATTTGCGTGTCAT |
| *GAPDH* | GTCAGCCGCATCTTCTTTTG | GCGCCCAATACGACCAAATC |
